# Molecular phylogenetic analysis of the *Amiota taurusata* species group within the Chinese species, with descriptions of two new species

**DOI:** 10.1093/jis/14.1.33

**Published:** 2014-01-01

**Authors:** Zhen-fang Shao, Tong Li, Jian-jun Jiang, Jin-ming Lu, Hong-wei Chen

**Affiliations:** 1 Department of Entomology, South China Agricultural University, Tianhe, Guangzhou, Guangdong, 510642, China; 2 Institute of Plant Protection, Henan Academy of Agricultural Science, Jinshui, Zhengzhou, Henan, 450002, China

**Keywords:** cryptic species, drosophilid, East Asia, mtDNA, taxonomy

## Abstract

The relationships among six species of the *Amiota taurusata* Takada, Beppu, & Toda (Diptera: Drosophilidae) species group were investigated based on DNA sequence data of the mitochondrial NADH dehydrogenase subunit 2 ( *ND2* ) gene, using three species of the genus *Amiota* as outgroups. A mitochondrial gene, cytochrome *c* oxidase I ( *COI* ), can be used to discriminate between species of the *taurusata* group. Two new species are described from South China: *A* . *protuberantis* Shao et Chen, sp. nov. and *A* . *shennongi* Shao et Chen, sp. nov. A key to all the species of the *taurusata* group based on morphological characters is provided.

## Introduction

The *Amiota taurusata* Takada, Beppu, & Toda (Diptera: Drosophilidae) species group was established by Chen and Toda (2001) based on a phylogenetic analysis using 31 adult male morphological characters. Until now, eight species have been reported in this group from East Asia ( Chen and Toda 2001 ; Chen et al. 2004 , 2005; Cao et al. 2008 ): *A. aquilotaurusata* Takada et al *.* , 1979, *A. asymmetrica*Chen et Takamori, 2005 ; *A. femorata*Chen et Takamori, 2005 , *A. sacculipes* Máca et Lin, 1993, *A. spinifemora* Li et Chen, 2008, *A. taurusata* Takada et al *.* , 1979, *A. vulnerabla*Chen et Zhang, 2004 , and *yixiangensis*Chen et Takamori, 2005 . Chen and Toda (2001) regarded the *taurusata* group as monophyletic on the basis of the hind femur basoventrally with a small, lobe-like flap (ch. 1; Figure 2 D in Chen and Toda 2001 ); hind tibia apicodorsally much extended flap (ch. 2; Figure 2 D in Chen and Toda 2001 ); hind first tarsomere dorsally expanded (ch. 3; Figure 2 D in Chen and Toda 2001 ); fourth tergite laterally broadened and protruded more than others (ch. 4; Figure 1 B in Chen and Toda 2001 ). However, Chen et al. (2004 , 2005) and Cao et al. (2008) found that the ch. 2 and ch. 3 are usually absent in some species; these two characters have been eliminated from the diagnosis criteria of the *taurusata* group.

**Figure 2.**
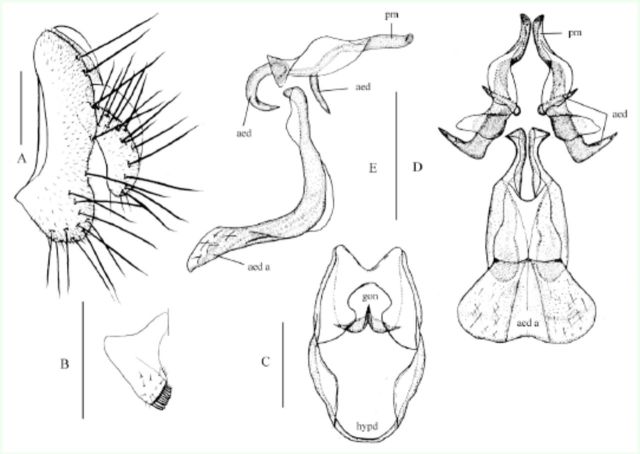
*Amiota shennongi* Shao et Chen, **sp. nov.** ♂: (A) Epandrium (epand) and circus, lateral view; (B) surstylus (sur) and tenth sternite (st 10), ventral view; (C) hypandrium and gonopod, ventral view; (D, E) paramere(s), aedeagus, and aedeagal apodeme, ventral and lateral views. Scale bars: 0.1 mm. High quality figures are available online.

**Figure 1.**
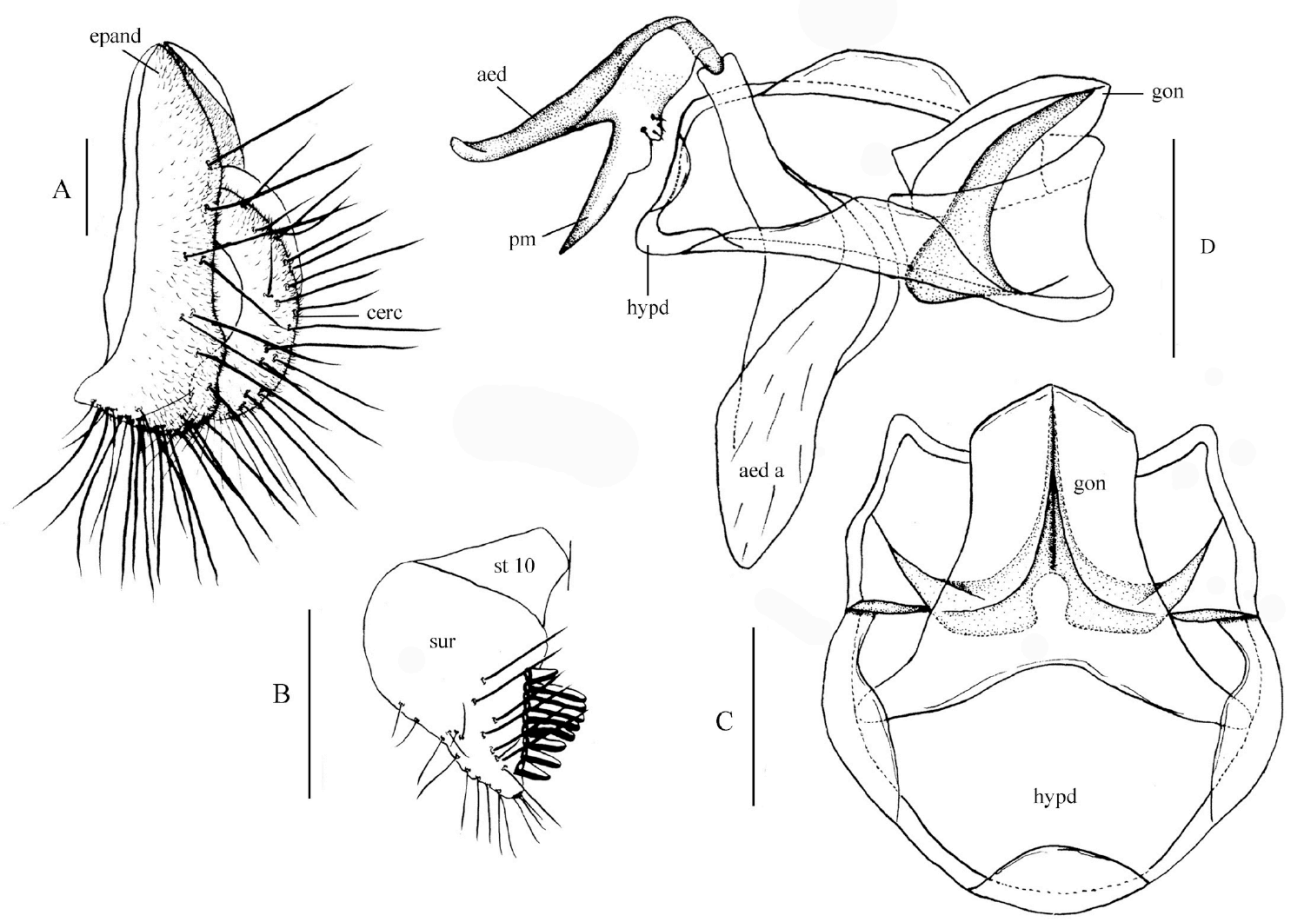
*Amiota protuberantis* Shao et Chen, **sp. nov.** ♂: (A) Epandrium (epand) and circus (cerc), lateral view; (B) surstylus (sur) and tenth sternite (st 10), ventral view; (C) hypandrium and gonopod, ventral view; (D) paramere, aedeagus, and aedeagal apodeme, lateral view. Scale bars: 0.1 mm. High quality figures are available online.

Recently, a molecular approach was used to uncover the relationship among the species in *Stegana* ( Li et al. 2010 ; Lu et al. 2011a , b), *Phortica* ( He et al. 2009b ; Cao et al. 2011 ), and *Paraleucophenga* ( Zhao et al. 2009 ), which are from genera of the subfamily Ste-ganinae. However, few related studies have been carried out in the genus *Amiota* . Chen and Toda’s (2001) phylogenetic analysis of the subgenus *Amiota* (currently the genus *Amiota* ) included the three species of this group mentioned above, the *taurusata* group, which is closely related to the *apodemata* , the *nagatai* , and the *sinuata* groups, but the relationships within this group were not resolved at all. In the present study, two new species of the *taurusata* group from China are described, and the relationships among the four known and two new species were investigated based on the DNA sequences of the mitochondrial NADH dehydrogenase subunit 2 ( *ND2* ) gene. Barcoding information on the mitochondrial cytochrome *c* oxidase I ( *COI* ) genes of most of the species is provided.

## Materials and Methods

### Materials

All materials were collected from tree trunks or around human eyes and preserved in 75% ethanol. A small piece of tissue was removed from the fly abdomen and used for the DNA extraction. The body and terminalia were dried and deposited in the Department of Entomology, South China Agricultural University, Guangzhou, China (SCAU). The definitions of measurements, indices, and abbreviations follow Zhang and Toda (1992) and Chen and Toda (2001) .

The information on the samples used in the molecular phylogenetic analyses is given in Table 1 . Six species of the *taurusata* group were employed in the molecular phylogenetic analysis, and three *Amiota* species of the genus *Amiota* from the *apodemata* , *nagatai* , and *sinuata* groups were used as outgroups.

**Table 1.**
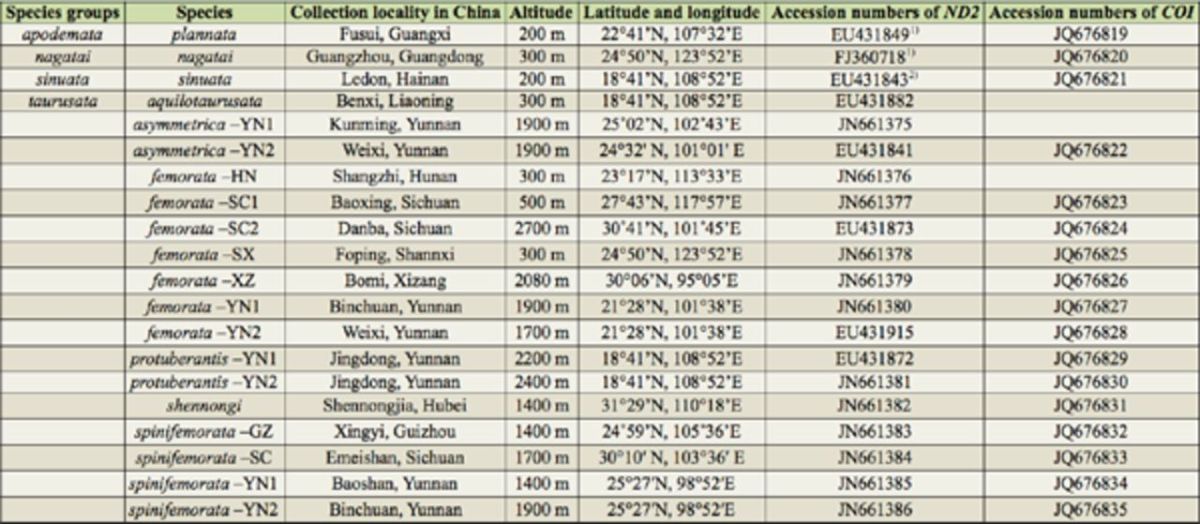
Data on samples for DNA sequencing and t he accession numbers of the *ND2* and *COI* sequences.

1) He et al. 2009a ; 2)mistaken as EU431907 in He et al. 2009a .

### Abbreviations

4c, third costal section between R2+3 and R4+5/M1 between r-m and dm-cu; 4v, M1 between dm-cu and wing margin/M1 between r-m and dm-cu; 5x, ac, third costal section between R2+3 and R4+5/fourth costal section; adf, longest dorsal branch of arista/width of first flagellomere; arb, dorsal branches/ventral branches of arista; avd, longest ventral branch/longest dorsal branch of arista in length; BL, body length; C, second costal section between subcostal break and R2+3/third costal section between R2+3 and R4+5; C3F, length of heavy setation in third costal section/length of the third costal section ch/o, maximum width of gena/maximum diameter of eye; CuA1 between dm-cu and wing margin/dm-cu between M1 and CuA1; dcl, anterior dorsocentral/posterior dorsocentral in length; dcp, length distance between ipsilateral dorsocentrals/cross distance between anterior dorsocentrals; flw, length/width of first flagellomere; FW/HW, frontal width/head width; M, CuA1 between dm-cu and wing margin/M1 between r-m and dm-cu; orbito, distance between proclinate and posterior reclinate orbitals/distance between inner vertical and posterior reclinate orbital; presctl, prescutellar/posterior dorsocentral in length; prorb, proclinate orbital/posterior reclinate orbital in length; rcorb, anterior reclinate orbital/posterior reclinate orbital in length; sctl, basal scutellar/apical scutellar in length; sctlp, distance between ipsilateral scutellars/cross distance between apical scutellars; sterno, anterior katepisternal/posterior katepisternal in length; THL, thorax length; vb, subvibrissal/vibrissa in length; WL, wing length; WW, wing width.

### DNA Extraction and Sequencing

Total DNA was extracted using a DNA extraction Kit (TIANGEN, www.tiangen.com ) according to the manufacturer’s protocol. The *ND2* and *COI* fragments were amplified with the primers listed in Table 2 . The PCR reactions consisted of an initial 4 min pre-denaturation at 94°C, followed by 30 cycles (30 sec of denaturation at 94°C, 1 min of annealing at 54°C for *ND2* and at 49°C for *COI* , and 1 min of extension at 72°C), and a final elongation for 5 min at 72°C. When possible, purified amplified products were directly run on an ABI 3730 sequencer; otherwise, they were cloned into the pMD18-T vector (TAKARA, www.takarabio.com ) and then sequenced. The related *ND2* sequences of *A. natagai* , *A. planate* , and *A. sinuata* were retrieved from the National Center for Biotechnology Information (NCBI).

**Table 2.**
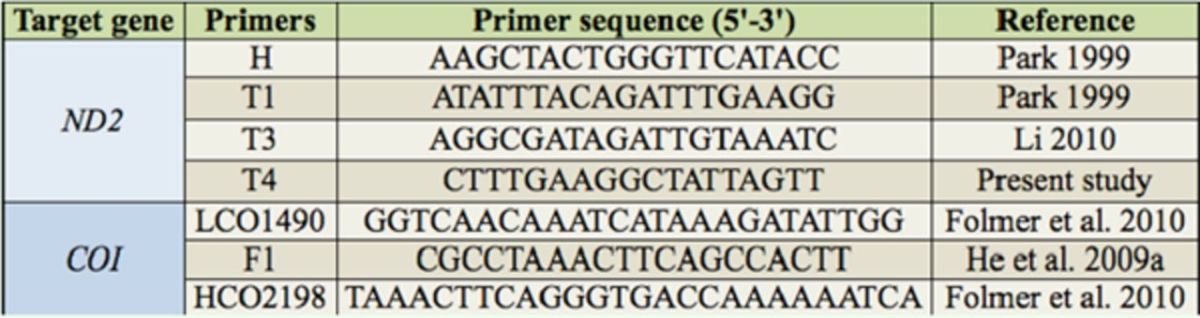
Primers used for PCR and sequencing.

### Phylogenetic analyses

The sequences were aligned by the Clustal W ( Thompson et al. 1994 ) method in MEGA 4.0 ( Tamura et al. 2007 ) with the default options and then adjusted manually. Because the substitution saturation masked the phylogenetic signal ( Lopez et al. 1999 ; Philippe and Froterre 1999 ), the method of Xia et al. (2003) was used to test the nucleotide substitution saturation in the program DAMBE 5.0.80 ( Xia and Xie 2001 ). The base compositions of these sequences were investigated using PAUP* version 4.0b10 ( Swofford 2001 ), and the c ^2^ test was used to evaluate the nucleotide composition homogeneity among them. Uncorrected *p* distance among taxa was estimated by MEGA 4.0 ( Tamura et al. 2007 ).

Phylogenetic relationships were constructed using the Bayesian inferring (BI) method in MrBayes 3.2.1 ( Huelsenbeck and Ronquist 2001 ; Ronquist and Huelsenbeck 2003 ). In the BI analyses, the data were partitioned by locus (1 data partition) and codon positions (3 data partitions). The nucleotide substitution models of BI analyses were selected by Modeltest 3.7 using the hierarchical likelihood ratio test (hLRT) criterion ( Posada and Crandall 1998 ). Two independent runs with 2,000,000 generations were implemented in parallel, and a sampling frequency of every 100 generations was employed. When the average deviation of split frequencies fell well below 0.01, the two runs were stopped. For each run, the 5,000 early-phase samples were discarded, and the remainder of the samples were used. A majority rule tree showing all the compatible partitions was obtained.

### Nomenclature

This publication and the nomenclature it contains have been registered in ZooBank. The LSID number is: urn:lsid:zoobank.org:pub:60353BF6-3506-4286-A8E9-FB72847CD3D9. It can be found online by inserting the LSID number after www.zoobank.org/ .

## Results

*Amiota taurusata* species group

### Diagnosis

Hind femur with small, lobe-like flap basoventrally; fourth tergite laterally broadened and protruded more than others (modified from Chen and Toda 2001 ; Figures 1B , 2D ). In the new species described, only characters that depart from the universal description (given by Chen and Toda (2001) and Chen et al. (2004 , 2005) for the subgenus *Amiota* ) are provided for brevity.

*Amiota protuberantis* Shao et Chen, sp. nov.( Figure 1 )

### Diagnosis

This species very similar to *A* . *femorata*Chen et Takamori, 2005 in hind tibia distinctly expanded on subapical part of dorsal surface; aedeagus bifurcated on basal 1/2, submedially slightly curved dorsad, separated from parameres in lateral view ( Figure 1 D).

### Description

Only important characters are given. Male and female: Frons, face, and clypeus nearly black. Ventral branches of arista distinctly shorter than 1/3 of dorsals in male, slightly shorter than 1/2 of dorsals in female. Palpus brown. Legs yellow except for dark brown on all femora in female or dark brown on femora of fore-and midlegs and distal half of hind femur in male. Male hindleg apicodorsally much extended like flap on tibia and dorsally slightly expanded on first tarsomere. Epandrium small, constricted more than 1/2 width mid-dorsally, with ca. 17 setae near posterior to ventral margins on each side ( Figure 1 A). Surstylus distally with numerous setae on outer surface and ca. 7 prensisetae ( Figure 1 B). Vertical lobe of gonopod apically round, without any processes. Parameres fused on basal 3/4, slightly sclerotized, as long as aedeagus, with ca. 9 sensilla subbasally ( Figure 1 C, D).

### Measurements

BL = 3.08 mm in the holotype (range in 3 *♂* and 2 *♀* paratypes: 2.60–3.16 mm in *♂* , 3.32– 3.40 mm in *♀* ), THL = 1.76 mm (1.56–1.72 mm in *♂* , 1.60–1.64 mm in *♀* ), WL = 2.60 mm (2.24–2.60 mm in *♂* , 2.80–2.88 mm in *♀* ), WW = 1.20 mm (1.00–1.20 mm in *♂* , 1.20– 1.32 mm in *♀* , arb = 6/4 (6/4–5), avd = 0.33 (0.33–0.57), adf = 1.00 (0.88–1.17), flw = 1.83 (1.50–2.00), FW/HW = 0.39 (0.36–0.44), ch/o = 0.08 (0.08–0.10), prorb = 0.94 (0.69– 1.00), rcorb = 0.50 (0.50–0.85), vb = 0.57 (0.57–0.63), dc1 = damaged, presct1 = 0.44 (0.44–0.60), sct1 = 1.20 (1.20–1.26), sterno = 0.78 (0.60–0.95), orbito = 1.00 (1.00–1.40), dcp = 0.38 (0.30–0.41), sct1p = 0.92 (0.72– 1.33), C = 2.05 (1.83–3.08), 4c = 1.73 (0.93– 1.78), 4v = 3.00 (2.50–3.00), 5x = 1.33 (1.16– 2.00), ac = 3.25 (3.25–5.33), M = 0.64 (0.50– 0.77), and C3F = 0.78 (0.78–0.83).

### Types

Holotype *♂* (SCAU, No. 121088), CHINA: Mt. Wuliang, Jingdong, Yunnan, 18°41’N, 108°52’E, altitude 2200 m a.s.l., 4.viii.2006, T Li. Paratypes: 3 *♂* , 2 *♀* (SCAU, No. 121089– 93), same data as the holotype.

### Etymology

From the Latin word protuberantis, referring to the hindleg tibia expanded on subapical part of dorsoposterior surface.

### Distribution

China (Yunnan).

*Amiota shennongi* Shao et Chen, sp. nov. ( Figure 2 )

### Diagnosis

This species very similar to *A* . *aquilotaurusata* Takada, Beppu et Toda, 1979 in that it has the same shape of the male terminalia. It differs by having the short process of aedeagus longer than 1/2 of long one ( Figure 2 D, E), the paramere thick rod-like, not expanded ( Figure 1 D, E).

### Description

Only important characters are given in here. Male: Frons, face, and clypeus nearly dark brown. Ventral branches of arista distinctly shorter than 1/3 of dorsals in male. Palpus brownish yellow. Legs entirely yellow; hind leg: tibia apicodorsally much extended flap, and first tarsomere dorsally expanded ( Chen and Toda 2001 ; Figure 2 D). Epandrium entirely separated into two lateral lobes, with about 15 setae near posterior to ventral margins per site ( Figure 2 A). Surstylus lacking pubescence, with finger-like process at posteroventral corner, and about nine prensisetae on distal margin ( Figure 2 B). Tenth sternite deeply constricted mid-ventrally, but not separated, entirely fused to surstyli laterally ( Figure 2 B). Anterior portion of hypandrium slightly broadened ( Figure 2 C). Aedeagus basally fused to paramere and deeply bifurcated, two processes of aedeagus nearly equilong ( Figure 2 D, E). Parameres slightly longer than aedeagus, round apically and expanded basally ( Figure 2 D, E).

### Measurements

BL = 2.88 mm in the holotype (3.00 mm in 1♂paratype), THL = 1.16 mm (1.27 mm), WL = 2.14 mm (2.44 mm), WW = 0.96 mm (1.24 mm), arb = 7/5 (5/4), avd = 0.29 (0.33), adf = 1.40 (1.20), flw = 2.40 (2.00), FW/HW = 0.46 (0.38), ch/o = 0.34 (0.24), prorb = 1.08 (0.87), rcorb = 0.75 (0.66), vb = 0.43 (0.50), dc1 = damaged (0.6), presct1 = 0.28(0.50), sct1 = 1.23 (1.09), sterno = 1.50 (0.75), orbito = 1.40 (3.30), dcp = 0.32 (0.36), sct1p = 1.33 (1.33), C = 2.57 (1.67), 4c = 1.27 (2.10), 4v = 3.55 (3.40), 5x = 0.63 (0.75), ac = 3.50 (5.25), M = 0.80 (0.80), and C3F = 0.63 (0.59).

### Types

Holotype *♂* (SCAU, No. 121094), CHINA: Dajiuhu, Shennongjia, Hubei, 31°29’N, 110°18’E, altitude 1400 m a.s.l., 31.vii.2004, HW Chen. Paratype: 1 *♂* (SCAU, No. 121095), same data as holotype.

### Etymology

Patronym, the name of Yandi, who was a man in an old Chinese story.

### Distribution

China (Hubei).

### Key to species of the *taurusata* group

1. Hind femur ventro-basally with nearly hyaline, small, lobe-like flap; fourth tergite laterally broadened and protruded more than others (the *taurusata* group). ………………………………………......2 Hind femur without any flap; fourth tergite neither broadened nor protruded more than others….............other *Amiota* species

2. Ventral branches of arista distinctly shorter than 1/2 of dorsals; all femora dark brown to black……………………….…...…….3 Ventral branches of arista as long as 1/2 of dorsals; all legs yellow….........................5

3. Hind first tarsomere not expanded dorsally………………………………….4 Hind first tarsomere expanded dorsally……….. *A. sacculipes* Máca et Lin

4. Vertical lobe of gonopod nearly triangular; aedeagus basally with 1 pair of slender processes… *A. femorata* Chen et Takamori Vertical lobe of gonopod nearly quadrate; aedeagus without any processes…………. ….. *A. protuberantis* Shao et Chen, sp. nov.

5. Hind tibia apicodorsally much extended like flap; hind first tarsomere expanded dorsally….................................................6 Hind tibia apicodorsally not extended like flap; hind first tarsomere not expanded dorsally………………………………….8

6. Short process of aedeagus shorter than 1/5 of long one……………………………….. …….. *A. taurusata* Takada, Beppu et Toda Short process of aedeagus slightly shorter or longer than 1/2 of long one..……….........7

7. Short process of aedeagus shorter than 1/2 of long one; paramere expanded to lobe-like….. *A. shennong* i Shao et Chen, sp. nov. Short process of aedeagus longer than 1/2 of long one; paramere expanded to lobe-like.. *………....A. aquilotaurusata* Takada, Beppu et Toda

8.Parameres nearly entirely sclerotized; gonopod nearly triangular………………... *………...A. asymmetrica* Chen et Takamori Paramereswith membranaceous part; gonopod nearly quadrate………………...9

9. Paramere entirely separated from aedeagus.. *……………A. yixiangna* Chen et Takamori Paramere basally fused to aedeagus………... …………… *A* . *vulnerabla* Chen et Zhang

### Molecular analysis

**Data set analysis**

The alignment of the sequences included 1026 base pairs for *ND2* and 684 for *COI.* There were end gaps in the *ND2* sequences of *A. spinifemorata* –GZ (sites 1–36), *A. spinifemorata* –YN2 (sites 1015–1026), and *A. shennongi* sp. nov. (sites 1015–1026). End gaps also existed in the *ND2* sequences (sites 149–151) of *A. femorata* –HN, *A. femorata* – SC2, *A. femorata* –SX, *A. femorata* –YN2, and *A. protuberantis* sp. nov.–YN2 as well as in the *ND2* sequences (sites 148–150) of *A. femorata* –SC1, *A. femorata* –YN2, *A. femorata* –XZ, and *A. protuberantis* sp. nov.–YN2. The *COI* sequences of some samples ( *A. aquilotaurusata* , *A. asymmetrica* –YN2, and *A. femorata* –HN) were not acquired, and end gaps existed in the *COI* sequences (sites 1–15) of *A. plannata.* There were 349 variable sites (of which 211 were parsimony informative sites) for *ND2* and 178 variable sites (of which 106 were parsimony informative sites) for *COI.* The nucleotide composition of *ND2* is shown in Table 3 . The sequences contained much higher AT content (82.8%) than GC content, especially at the third codon positions (95.4%). The c ^2^ test revealed that the nucleotide composition among the taxa was not het-heterogeneous.

Regardless of whether the analysis was performed with the combined or separated codon position data for *ND2* , the test of substitution saturation revealed that the observed substitution saturation index ( *Iss* ) was significantly lower than the corresponding critical substitution saturation index ( *Iss.c* ) for both the symmetrical and asymmetrical trees, indicating that there was little saturation in these sequences ( Table 4 ).

**Table 3.**
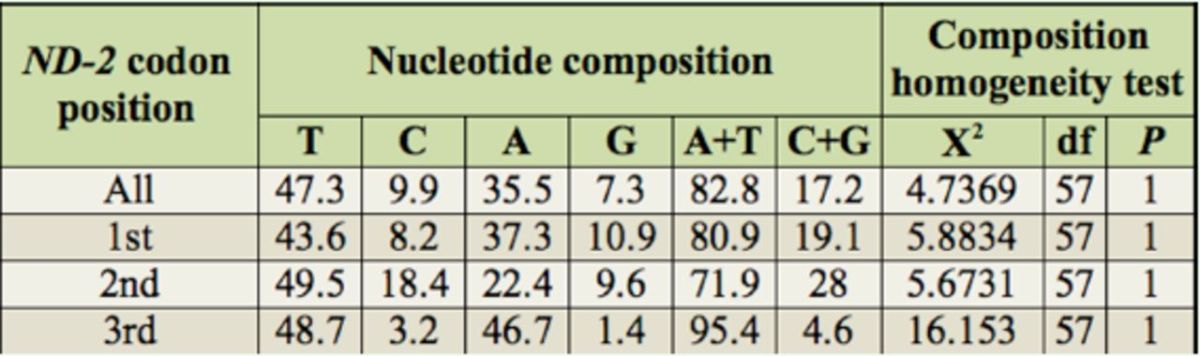
Results of nucleotide composition and composition homogeneity test.

**Table 4.**
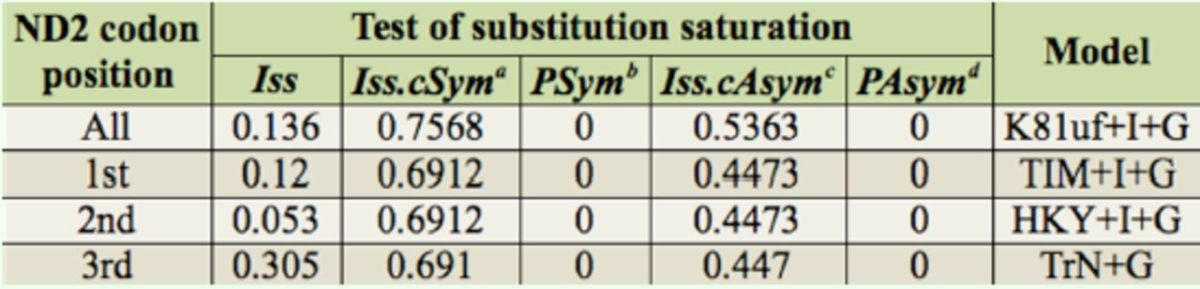
Results of substitution saturation tests and model selection.

*^a^* Index of substitution saturation assuming a symmetrical true tree.

*^b^* Probability of a significant difference between *Iss* and *Iss.cSym* (two-tailed test).

*^c^* Index of substitution saturation assuming an asymmetrical true tree.

*^d^* Probability of a significant difference between *Iss* and *Iss.cAsym* (two-tailed test).

Table 5 shows the uncorrected pairwise divergence for the *ND2* and *COI* sequences in the *taurusata* group, excluding the p-distance for *COI* of *A. aquilotaurusata* , *A. asymmetrica* –YN1, and *A. femorata* –HN. The interspecific genetic divergence for *ND2* in the *taurusata* group ranged from 0.0294 ( *A. femorata* vs. *A. protuberantis* sp. nov.) to 0.1049 ( *A. aquilotaurusata* vs. *A. spinifemorata* ), and for *CO1* it ranged from 0.0207 ( *A. femorata* vs. *A. protuberantis* sp. nov.) to 0.0841 ( *A. asymmetrica* vs. *A. protuberantis* sp. nov.). The intraspecific genetic divergences for *ND2* and *COI* were calculated for *A. asymmetrica* (0.0021 for *ND2* ), *A. protuberantis* sp. nov. (0.0072 for *ND2* , 0.0015 for *COI* ), *A. spinifemorata* (0.0041 to 0.0185 for *ND2* , 0.0073 to 0.0088 for *COI* ), and *A. femorata* (0.0010 to 0.0474 for *ND2* , 0.0000 to 0.0336 for *COI* ).

**Table 5.**
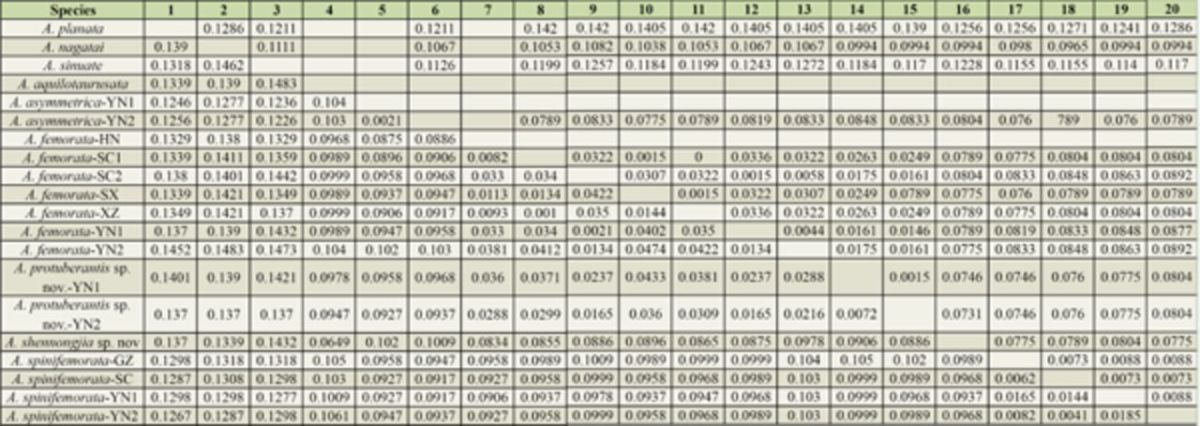
Uncorrected pairwise p-distance among the *ND2* and *COI* sequences of the *taurusata* species group. The matrix in the lower left shows the uncorrected pairwise p-distance among the *ND2* sequences; the matrix in the upper right shows the uncorrected pairwise pdistance among the *COI* sequences.

### Phylogenetic analysis

**Figure 3.**
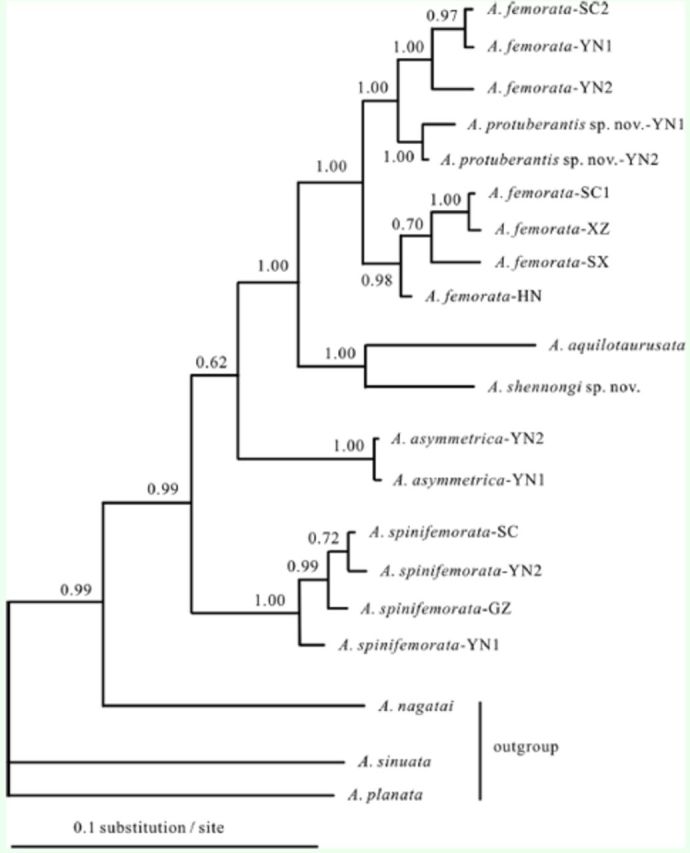
Bayesian tree of the *taurusata* group deduced from the *ND2* sequences using the site-specific mode numbers. These numbers are beside the nodes and are the posterior probabilities. High quality figures are available online.

The Bayesian tree for *ND2* lent good support for the monophyly of the *taurusata* group with respect to the outgroups (posterior probabilities (PP) = 0.99) ( Figure 3 ). Samples from different geographical areas of *A. protuberantis* , *A. asymmetrica* , and *A. spinifemorata* clustered as a monophyletic lineage, while samples of *A. femorata* were rendered paraphyletic with respect to *A. protuberantis* . *A. spinifemorata* first diverged in the *taurusata* group and was then followed by *A. asymmetrica* . The other four species grouped into a robust supported group (PP = 1.00). *A* . *shennongi* sp. nov. and *A* . *aquilotaurusata* showed a sibling relationship (PP = 1.00) in agreement with their high similarity in morphological characters. Samples of *A. femorata* diverged into two highly supported clusters. One consisted of *A. femorata* (HN, SC1, SX, and XZ) (PP = 0.98); the other consisted of *A. femorata* (SC2 and YN1–2) (PP = 1.00) and clustered with *A. protuberantis* sp. nov. (PP = 1.00).

## Discussion

A phylogenetic tree of the *taurusata* group was constructed using mitochondrial *ND2* sequences. As negative results were obtained in the tests of nucleotide composition heterogeneity and substitution saturation, the conclusions of the phylogenetic analyses should be accepted. The monophyly of the *taurusata* group was strongly supported in the molecular phylogenetic analyses, and the relationships within this group were almost resolved. However, the unstable position of *A. asymmetrica and A. spinifemorata* was not resolved, even when using a site-specific model for Bayesian inference. To fully resolve the phylogenetic relationship in the *taurusata* group, multiple loci or more species in the analyses are necessary.

The *ND2* divergence matrix was provided for the *taurusata* group. The interspecific genetic divergence in the *taurusata* group ranged from 0.0294 to 0.1049, and the intraspecific genetic divergence ranged from 0.0010 to 0.0474. The geographical samples of *A. asymmetrica* , *A. protuberantis* sp. nov., and *A. spinifemorata* formed highly-supported monophyletic groups in the phylogenetic tree, and intraspecific genetic divergence within them was much less than interspecific genetic divergence in the *taurusata* group. In addition, no diagnostic morphological character was found to distinguish the geographical samples of these species, indicating that they should be considered conspecific. However, *A. femorata* diverged into two clusters, and classified characters in its morphology were missing. The three haplotypes of *A. femorata* , i.e., SC2, YN1, and YN2, clustered with *A. protuberantis* sp. nov. This relationship could be attributed to stochastic lineage sorting and/or hybridization. The genetic divergence for *ND2* within the two clusters ranged from 0.0010 to 0.0474, and the mean divergence between them was 0.0392. Assuming the observed divergence range (0.0294 to 0.1049) reflects the real intraspecific variations in the *taurusata* group, there likely are cryptic species in *A. femorata* samples.

Recent work suggests that cytochrome c oxidase I ( *COI* ) might serve as a DNA barcode for the identification of animal species ( Brown et al. 2003 ; Foster et al. 2004; Barrett and Hebert 2005 ; Cardoso and Vogler 2005 ; Hogg and Hebert 2005; Monaghan et al. 2005; Vences et al. 2005 ; Ward et al. 2005 ). This gene region is easily recovered and provides good resolution, as evidenced by the deep sequence divergences among 13,000 closely related pairs of animal species (Hebert et al. 2003b). In this study, a 684 bp region of *COI* was acquired, and it showed that *COI* differences between most of the species far exceeded those within species. The interspecific genetic divergence in the *taurusata* group ranged from 0.0207 to 0.0841, and the intraspecific genetic divergence ranged from 0.0000 to 0.0336. An overlapping area existed between the intraspecific and interspecific genetic divergence. The intraspecific genetic divergences within *A. protuberantis* sp. nov. (0.0015) and *A. spinifemorata* (0.0073 to 0.0088) were much lower than the minimum interspecific genetic divergence (0.0207) and the mean intraspecific variability for Diptera (1.3 ± 1.6%) ( Meier 2008 ), indicating that they should be considered conspecific. This result is consistent with the *ND2* result and the morphology analysis. The observed divergence of the two clusters of *A. femorata* was 0.0322, which is greater than the minimum interspecific genetic divergence but lower than the minimum interspecific genetic divergence for Diptera (5.9 ± 4.1%) ( Meier 2008 ). *A. protuberantis* sp. nov. was identified as the sister-species of *A. femorata* , and the p-distance ranged from 0.0146 to 0.0263. Because of the limit coming from the number and distribution of samples, there likely are cryptic species in the two clusters, which is consistent with the *ND2* result. It is important to include samples from a wider geographical range in future studies to determine if the two clusters represent morphologically cryptic species. Further samples are also needed for an evaluation of the morphological variability revealed in the results.

### Biogeographical implications

All the members of *A. spinifemorata* and *A. asymmetrica* were found in southwestern China. According to the phylogenetic analyses, the two species diverged from the *taurusata* group prior to *A. shennongi* sp. nov. and *A. aquilotaurusata* , which were found in central and northeast China. It may indicate that the founder of the *taurusata* group arose in southwestern China, undergoing some differentiation before the expansion into the central and northern areas. In the zones of low and high elevation, *A. femorata* can be distributed between one cluster ( *A. femorata* –HN, SC1, SX, excluding *A. femorata* –XZ) mainly at low elevations (ca. 300–500 m a.s.l.) and another cluster ( *A. femorata* –SC2, YN1, and YN2) at high elevations (ca. 1700–2700 m a.s.l.) that clusters with *A. protuberantis* sp. nov. This result may indicate that some individuals underwent heteromorphosis to different extents following the expansion of *A. femorata* from low elevations into high elevations, and that then *A. protuberantis* sp. nov. was found.
